# Modelling lowering of raised blood pressure in pregnancy to reduce pre-eclampsia: secondary analysis of data from prospective cohort studies

**DOI:** 10.1136/bmjmed-2025-001631

**Published:** 2026-03-27

**Authors:** Alan Wright, Argyro Syngelaki, David Wright, Peter von Dadelszen, Kypros H Nicolaides, Laura A Magee

**Affiliations:** 1 Institute of Health Research, University of Exeter, Exeter, UK; 2 King’s College Hospital NHS Foundation Trust, London, UK; 3 Department of Women and Children’s Health, King’s College London, London, UK; 4 Fetal Medicine Research Institute, King’s College Hospital, London, UK

**Keywords:** Hypertension, Obstetrics

## Abstract

**Objective:**

To determine whether antihypertensive treatment of blood pressure levels of <140/90 mm Hg can reduce the incidence of pre-eclampsia.

**Design:**

Secondary analysis of data from prospective cohort studies.

**Setting:**

Three prospective screening studies of women who attended routine hospital maternity visits at 11-13 weeks' gestation, 1 February 2010 to 31 December 2016. Participants were from seven secondary care institutions in England.

**Participants:**

54 422 pregnancies screened at 11-13 weeks' gestation for pre-eclampsia and with blood pressure values available, that resulted in a liveborn or stillborn infant at ≥24 weeks' gestation.

**Main outcome measures:**

Incidence of pre-eclampsia (overall, and at preterm or term gestational ages), according to modelled blood pressure lowering.

**Results:**

The study population was ethnically diverse (17.3% black participants, 7.8% from South or East Asia, and 2.6% self-identified with more than one ethnic group). The Fetal Medicine Foundation competing risks model was used to calculate the expected risk of pre-eclampsia, based on maternal characteristics, mean arterial pressure, uterine artery pulsatility index, and placental growth factor. The expected risk of pre-eclampsia was used to calculate the expected incidence of pre-eclampsia. Reducing diastolic blood pressure from >85 mm Hg to a target of 85 mm Hg would mean that 4.8% of women would be offered antihypertensive drugs, with a potential relative risk reduction of 21.4% (absolute reduction of 2.9%) in any pre-eclampsia and 28.3% (absolute reduction of 1.4%) reduction in preterm pre-eclampsia. By reducing diastolic blood pressure from >80 mm Hg to a target of 80 mm Hg, 13.2% of women would receive antihypertensive drugs, with a potential relative risk reduction of 26.0% (absolute reduction of 2.3%) in any pre-eclampsia and 33.8% (absolute reduction of 1.0%) reduction in preterm pre-eclampsia. By reducing diastolic blood pressure from >75 mm Hg to a target of 75 mm Hg, 29.5% of women would receive antihypertensive drugs, with a potential relative risk reduction of 32.8% (absolute reduction of 2.1%) in any pre-eclampsia and 41.6% (absolute reduction of 0.8%) in preterm pre-eclampsia.

**Conclusions:**

Lowering blood pressure from early pregnancy may reduce preterm and term pre-eclampsia. This finding requires evaluation in a definitive randomised trial.

**WHAT IS ALREADY KNOWN ON THIS TOPIC**

- Most clinical practice guidelines recommend antihypertensive treatment for blood pressure levels of ≥140/90 mm Hg, and further reductions in early pregnancy might reduce the risk of pre-eclampsia and other adverse pregnancy outcomes
- Different blood pressure thresholds for intervention could be appropriate based on different maternal characteristics

**WHAT THIS STUDY ADDS**

- Adjusting for factors that affect the risk of pre-eclampsia and blood pressure level, this modelling study suggested that lowering blood pressure from early pregnancy may reduce preterm and term pre-eclampsia by up to almost half

**HOW THIS STUDY MIGHT AFFECT RESEARCH, PRACTICE, OR POLICY**

- A large definitive intervention trial is needed to inform clinical practice
- The number needed to treat to benefit those with raised blood pressure may vary from 15.6 currently (for diastolic blood pressure >90 mm Hg at 11-13 weeks), to 36 (for diastolic blood pressure >85 mm Hg), and up to 48 (for diastolic blood pressure >75 mm Hg)

## Introduction

 Outside of pregnancy, higher blood pressure levels are associated with a greater risk of cardiovascular disease, but a clear threshold is needed to guide clinical practice. In 2017, the American College of Cardiology and American Heart Association revised the definition of hypertension outside of pregnancy to: stage 2 hypertension, corresponding to the traditional definition of systolic blood pressure ≥140 mm Hg or diastolic blood pressure ≥90 mm Hg; stage 1 hypertension, at a systolic blood pressure of 130-139 mm Hg or a diastolic blood pressure of 80-89 mm Hg; raised blood pressure, as a systolic blood pressure of 120-129 mm Hg (with associated diastolic blood pressure of <80 mm Hg); and normal blood pressure, defined as a systolic blood pressure of <120 mm Hg and diastolic blood pressure of <80 mm Hg.^1^ These definitions are similar to the European Society of Cardiology guidelines: hypertension is defined as blood pressure level of ≥140/90 mm Hg in the office, raised blood pressure is considered to be 120/70 to <140/90 mm Hg, and normal blood pressure is <120/70 mm Hg.^2^

Since the publication of two seminal randomised trials of blood pressure control for women with chronic^3 4^ or gestational^3^ hypertension, the International Society for the Study of Hypertension in Pregnancy and most clinical practice guidelines recommend antihypertensive treatment for stage 2 hypertension in pregnancy.^5^ In pregnancy, raised blood pressure in the first trimester is associated with an increased risk of adverse outcomes.^6^ Normalising blood pressure in early pregnancy might reduce the risk of placental maldevelopment and the associated risk of developing pre-eclampsia, fetal growth restriction, and other adverse pregnancy outcomes.^7^ To justify evaluating an intervention for a fixed blood pressure threshold with antihypertensive treatment, however, a fixed cut-off value for first trimester blood pressure should meaningfully distinguish between those who are and those who are not at a greater risk of adverse pregnancy outcomes. This ability has not been shown by systematic review of studies of fixed blood pressure thresholds in the first^8^ or second half of pregnancy.^9^ Some evidence exists, however, indicating that different thresholds for intervention may be appropriate according to different maternal characteristics, particularly those who have underweight or are parous with no history of pre-eclampsia, who comprise at least half of the maternity population.^10^

In a large unselected maternity population who were ethnically and socioeconomically diverse, we undertook a modelling study to evaluate the potential effect on the incidence of pre-eclampsia of lowering raised blood pressure in early pregnancy. The blood pressure level was based on a value adjusted for relevant factors influencing blood pressure and the risk of pre-eclampsia (eg, body weight) from early pregnancy, accounting for other risk factors for pre-eclampsia.

## Materials and methods

### Study population

We undertook a secondary analysis of data from three prospective screening studies of women who attended routine hospital maternity visits at 11-13 weeks' gestation, from 1 February 2010 to 31 December 2016.^11^ Data for sex were taken from information in the studies, rather than from patient-reported gender. Participants were pregnant women and other pregnant people. All participants provided written informed consent for participation.

In this analysis, we included women with a singleton pregnancy who gave birth to a liveborn or stillborn infant at ≥24 weeks' gestation, and who underwent first trimester screening for pre-eclampsia and had blood pressure values available; women with chronic hypertension were included as their management would be different (see Analysis section). We excluded pregnancies with aneuploidy, major fetal abnormalities, and those ending in miscarriage or termination before 24 weeks' gestation.

The Fetal Medicine Foundation multivariable competing risks model was applied for assessment of the risk of pre-eclampsia at 11-13 weeks' gestation. This model incorporates maternal factors and biomarkers, and is based on a survival time model for the gestational age at delivery with pre-eclampsia.^12^ An assumption is that if the pregnancy were to continue indefinitely, all women would develop pre-eclampsia, and whether or not they develop pre-eclampsia before a specific gestational age depends on competition between birth before or after pre-eclampsia develops. A gaussian model for gestational age at delivery was chosen, based on model fit and interpretability. In pregnancies at low risk of pre-eclampsia, maternal factors and biomarkers shift the gestational age distribution to the right, meaning that most births will occur before pre-eclampsia develops. In pregnancies at high risk of pre-eclampsia, maternal factors and biomarkers shift the gestational age distribution to the left, so the smaller the mean gestational age of this distribution, the higher the risk for pre-eclampsia. Pre-eclampsia risk scoring is continuous and the screen positive rate can be adjusted to the desired population risk cut-off value. The model has been externally validated in a mixed UK and European population,^13^ as well as in populations in Asia,^14^ Spain,^15^ and Denmark.^16^ The model is available at https://fetalmedicine.org/.

The variables used in the first trimester Fetal Medicine Foundation competing risks model were: maternal personal characteristics and medical history^17^; weight and height; mean arterial pressure by validated automated devices and standardised protocol, which involved taking a minimum of two blood pressure measurements from each arm^18^; left and right uterine artery pulsatility index by transabdominal colour Doppler ultrasound and calculation of mean uterine artery pulsatility index^19^; and maternal serum concentrations of placental growth factor, measured by one of two automated devices (Delfia Xpress analyser, PerkinElmer Life and Analytical Sciences, Waltham, USA or Brahms Kryptor analyser, Thermo Fisher Scientific, Hennigsdorf, Germany). Gestational age was determined by measurement of fetal crown-rump length. The pre-eclampsia risk calculator is available online through a standalone calculator (https://fetalmedicine.org/research/assess/preeclampsia/).

### Antihypertensive treatment

The current approach to treatment of high blood pressure is to offer antihypertensive drug treatment to women with a diastolic blood pressure of ≥90 mm Hg to achieve a target diastolic blood pressure of 85 mm Hg,^20^ based on the results of the Control of Hypertension in Pregnancy Study (CHIPS)^3^ and the Chronic Hypertension and Pregnancy (CHAP) trial.^4^ Because these trials were published in 2015 and 2022, respectively, blood pressure control to <140/90 mm Hg in pregnancy has been advocated only in the past 5-10 years.^21^

We did not restrict our investigation to the use of a specific antihypertensive agent. Labetalol is generally the antihypertensive agent of choice in pregnancy for non-severe hypertension, but a wide range of inexpensive, widely available options also exist. (Only renin-angiotensin-aldosterone system blockers are contraindicated for use in pregnancy, based on their fetotoxicity.) In a network meta-analysis of 61 informative randomised trials, all commonly prescribed antihypertensive drugs (labetalol, other beta blockers, methyldopa, calcium channel blockers, and mixed or multi-drug treatments) versus placebo or no treatment, halved the risk of severe hypertension (range 30-70%), but few clear differences were found between treatments and the 95% credible intervals were wide. Labetalol decreased proteinuria, presumably of pre-eclampsia (odds ratio 0.73, 95% credible interval 0.54 to 0.99), and fetal or newborn death (odds ratio 0.54, 0.30 to 0.98) compared with placebo or no treatment, and decreased proteinuria compared with methyldopa (odds ratio 0.66, 0.44 to 0.99) and calcium channel blockers (odds ratio 0.63, 0.41 to 0.96). Also, antihypertensive drugs in pregnancy are associated with a low rate of adverse effects (about 2%); no difference has been found between antihypertensive drugs and placebo or no treatment, or between other antihypertensive drugs, although the 95% confidence intervals are wide .^22 23^

Antihypertensive treatment in pregnancy lowers blood pressure, with the magnitude of effect dependent on the target blood pressure level. The goal of lowering diastolic blood pressure by 5 mm Hg, to a target diastolic blood pressure of 85 mm Hg, was achieved in the CHIPS trial.^3^ Lowering blood pressure by this magnitude was sufficient to favourably affect maternal outcomes (ie, severe hypertension and severe features of pre-eclampsia), without having a negative effect on fetal growth. Blood pressure was lowered by 5.8 (systolic)/4.6 (diastolic) mm Hg in CHIPS and by 3.1 (systolic)/2.4 (diastolic) mm Hg in the CHAP trial. The severe features of pre-eclampsia were 38% of control in CHIPS (based on platelet count <100×10^9^/L) and 80% in the CHAP trial. This finding suggests that for about double the blood pressure reduction in CHIPS, halving of the incidence of severe features of pre-eclampsia was achieved.

### Outcomes

Outcome data were collected from hospital maternity or general medical practitioners' records. The key outcome was pre-eclampsia, defined by the American College of Obstetricians and Gynaecologists as chronic or gestational hypertension, with development of one or more of: new onset proteinuria, serum creatinine concentration >97 µmol/L in the absence of underlying renal disease, levels of serum transaminases more than twice normal (≥65 IU/L for our laboratory), platelet count <100 000/µL, headache or visual symptoms, or pulmonary oedema.^24^ Gestational hypertension was new onset hypertension at ≥20 weeks' gestation in a previously normotensive woman.^24^ Preterm pre-eclampsia was pre-eclampsia with birth at <37 weeks' gestation and term pre-eclampsia with birth at ≥37+0 weeks' gestation.

Other outcomes were preterm birth (<37+0 weeks' gestation), small-for-gestational age infants (birth weight <10th or <3rd centile for gestational age, based on the Fetal Medicine Foundation fetal and neonatal weight charts),^11^ stillbirth (infant born at ≥24+0 weeks' gestation), neonatal death (up to 28 days of life), and admission to the neonatal intensive care unit. Chronic hypertension was systolic blood pressure ≥140 mm Hg or diastolic blood pressure ≥90 mm Hg, or both, at least twice, four hours apart, reported before pregnancy or at <20 weeks' gestation.^25^

Contemporaneous management of pregnancy hypertension (and its prevention) was according to local policy, based on national guidance. Assessing the number and nature of clinical risk factors in early pregnancy identifies about 10% of women who have an increased risk of pre-eclampsia. Typically, women with at least one high risk factor (ie, hypertensive disease in previous pregnancy, chronic hypertension, diabetes mellitus, chronic kidney disease, and autoimmune disease) or two moderate risk factors (ie, first pregnancy, age >40 years, body mass index at first visit >35, interval between pregnancies >10 years, and family history of pre-eclampsia) are considered to have a high risk of pre-eclampsia.^20^ Those at increased risk of pre-eclampsia are offered low dose aspirin (81-162 mg/day). Antihypertensive treatment was started at a blood pressure level of ≥150/100 mm Hg, without specifying a target blood pressure,^26^ until June 2019, when a lower treatment threshold of 140/90 mm Hg (and target of ≤135/85 mm Hg) was recommended.^27^ Pre-eclampsia was expectantly managed until 37+0 weeks throughout the study period.^28^ This analysis was about prediction of pre-eclampsia, and not core maternal outcomes associated with pre-eclampsia after the condition developed.^29^

### Analysis

We used descriptive analyses for the cohort overall for baseline maternal characteristics, blood pressure level, and pregnancy outcomes. Continuous variables are summarised as median (interquartile range) and categorical variables as number (%).

The Fetal Medicine Foundation competing risks model was used to calculate the expected risk of any pre-eclampsia, pre-eclampsia at <32 weeks, pre-eclampsia at <34 weeks, pre-eclampsia at <37 weeks, and pre-eclampsia at ≥37 weeks, for each pregnancy, and the total expected number of patients with pre-eclampsia for the whole population was derived by summing the decimal risks. The Fetal Medicine Foundation model is based on maternal and pregnancy characteristics, mean arterial pressure, uterine artery pulsatility index, and placental growth factor.

Model calibration was checked by calibration plots. The observed incidence of pre-eclampsia was plotted against predicted pre-eclampsia incidence from risk, by grouping data into bins according to the risk of pre-eclampsia. We quantified calibration by the estimated intercept from a logistic regression of incidence of pre-eclampsia on the logit of pre-eclampsia risk, with the slope fixed at 1, and by the estimated slope of the regression line of the logistic regression of incidence on the logit of pre-eclampsia risk. For perfectly calibrated risks, the intercept should be 0 and slope should be 1.0.

We modelled the current treatment approach (ie, offering antihypertensive drug treatment to women with a diastolic blood pressure of ≥90 mm Hg to achieve a target diastolic blood pressure of 85 mm Hg).^20^ We also modelled the effect of antihypertensive treatment to reduce diastolic blood pressure levels of >85, 80, or 75 mm Hg to achieve target diastolic blood pressure values of 85, 80, or 75, respectively. This approach means that if diastolic blood pressure were >85 mm Hg (with or without antihypertensive treatment), more women with chronic hypertension would be treated with antihypertensive drugs, and those on treatment would receive more drug treatment, because their target blood pressure would be lower (80 or 75 mm Hg). Diastolic blood pressure was chosen as our focus because it better reflects hypertension in the young,^30^ was not inferior to systolic blood pressure in the CHAP trial,^31^ and for ease of clinical translation, as in CHIPS.^3^ In our approach, diastolic blood pressure values exceeding a specified threshold were fixed at that threshold, and systolic blood pressure values were simulated with a predefined model (online supplemental table S1), under the assumption that a reduction in diastolic blood pressure would correspond to a reduction in systolic blood pressure.

**Figure 1:**
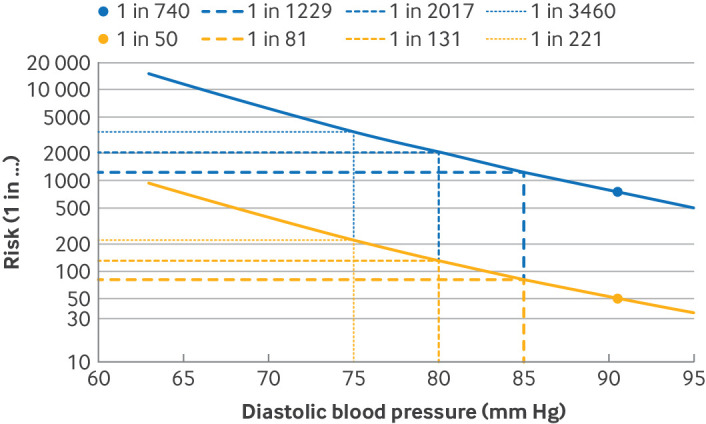
Example of individual pre-eclampsia risk profiles by diastolic blood pressure, for high risk (yellow) and low risk (blue) individuals

Mean arterial pressure and the risk of delivery with pre-eclampsia were then recalculated, and these updated risk estimates were used to model the incidence of pre-eclampsia. Figure 1 illustrates the effect of modifying diastolic blood pressure on the estimated risk for individuals with a high risk and low risk of pre-eclampsia; as diastolic blood pressure falls, the risk of pre-eclampsia falls (ie, one in a large number) for women at high risk or low risk of pre-eclampsia. The results are presented as relative risk reduction for preterm, term, and all pre-eclampsia, based on a comparison between the modelled pre-eclampsia event rates under antihypertensive treatment and the observed pre-eclampsia event rates. The number needed to benefit was calculated as the inverse of the absolute risk reduction associated with antihypertensive treatment.

We made these assumptions: reducing the calculated risk of pre-eclampsia (by reducing blood pressure) would directly affect the incidence of pre-eclampsia; treatment with aspirin had no effect on preterm pre-eclampsia (given the poor screening performance of clinical risk factors alone,^17^ poor adherence to the recommendation to offer aspirin to women with an increased risk by clinical risk factors,^32^ lack of an interaction between aspirin treatment and baseline risk of pre-eclampsia,^33^ and lack of effect of aspirin on preterm pre-eclampsia in women with chronic hypertension^33^); no interactions between aspirin and other treatments (including antihypertensive treatment); and diastolic blood pressure achieved with antihypertensive treatment (of any commonly used agent in pregnancy) would be associated with a similar pre-eclampsia risk to one that was achieved spontaneously. Furthermore, we assumed that greater falls in blood pressure would achieve greater falls in pre-eclampsia, and that adverse effects would be negligible (see Antihypertensive treatment section). All analyses were performed with R statistical software package (version 4.3.3).

### Patient and public involvement

Patients and/or the public were not involved in the design, or conduct, or reporting, or dissemination plans of this research because this was a modelling study.

## Results

We screened 61 174 singleton pregnancies at 11-13 weeks' gestation from our potential study population from England, Spain, Belgium, Italy, and Greece. Only 54 422 pregnancies had measurements of systolic and diastolic blood pressure and were included in the analysis. All participants were from the seven secondary care institutions in England.

Table 1 shows that median age was 31.3 years and participants were from diverse ethnic groups. We found that 47.7% of individuals had overweight or obesity. Less than 5% of women reported chronic hypertension, pre-gestational diabetes, or systemic lupus erythematosus. A small proportion of participants (8.5%) were smokers and 4.4% reported a family history of pre-eclampsia in their mother or sister. Conception was by assisted means in 3.5% of women, and 52.9% of women were parous, with 3.1% overall reporting previous pre-eclampsia when seen at about 13 weeks' gestation in the next pregnancy, an average of 2.9 years later.

**Table 1:** Baseline and pregnancy characteristics of study population

Characteristics	All pregnancies (n=54 422)
Baseline characteristics:	
Median (IQR) age (years)*†	31.30 (27.00-35.00)
Ethnic group:*†	
White	39 362 (72.33)
Black	9435 (17.34)
South Asian	3007 (5.53)
East Asian	1217 (2.24)
Mixed	1401 (2.57)
Median (IQR) body mass index:*†‡	24.70 (22.00-28.60)
Overweight (25.0-29.9)	15 274 (28.07)
Obese (≥30.0)	10 698 (19.66)
≥35.0	3883 (7.13)
Medical history:	
Chronic hypertension*	728 (1.34)
Diabetes mellitus type 1*	197 (0.36)
Diabetes mellitus type 2*	270 (0.50)
Systemic lupus erythematosus or antiphospholipid antibody syndrome*	106 (0.19)
Smoker*†	4603 (8.46)
Family history of pre-eclampsia:*	
In mother	2131 (3.92)
In sister	283 (0.52)
Pregnancy characteristics:	
Assisted method of conception*†	1890 (3.47)
Parity:*†	
Nulliparous	25 644 (47.12)
Parous, no previous pre-eclampsia	27 089 (49.78)
Parous, previous pre-eclampsia	1689 (3.10)
Pre-eclampsia screening:	
Median (IQR) gestational age at screening (weeks)	12.70 (12.30-13.10)
Median (IQR) interval between pregnancies (years)	2.90 (1.80-4.90)
Blood pressure:	
Systolic <120 mm Hg and diastolic <80 mm Hg	32 778 (60.23)
Systolic 120-129 mm Hg and diastolic blood <80 mm Hg	11 489 (21.11)
Systolic 130-139 mm Hg or diastolic 80-89 mm Hg	8449 (15.52)
Systolic ≥140 mm Hg or diastolic ≥90 mm Hg	1706 (3.13)
Aspirin	1210 (2.22)
Pregnancy outcomes:	
Median (IQR) gestational age at delivery (weeks)	40.00 (39.00-40.90)
Preterm birth:	3041 (5.58)
Iatrogenic	1263 (2.32)
Spontaneous	1778 (3.26)
Preterm pre-eclampsia	453 (0.83)
Term pre-eclampsia	1140 (2.09)
Caesarean section	14 700 (27.01)
Median (IQR) birth weight (g):	3400.00 (3060.00-3720.00)
Birth weight <10th centile	7276 (13.37)
Birth weight <3rd centile	3171 (5.83)
Stillbirth	185 (3.40/1000)
Neonatal death	23 (0.42/1000)
Admission to neonatal intensive care unit§	3089 (5.67)

Values are number (%) unless indicated otherwise.

* These characteristics are associated with an increased risk of pre-eclampsia according to the Fetal Medicine Foundation competing risks model.

† These characteristics are associated with mean arterial blood pressure in women who do not have an increased risk of pre-eclampsia.^6^

‡ Institute of Medicine body mass index criteria were used to define overweight (25.0-29.9) and obesity (≥30).

§ Data on admission to the neonatal unit were systematically collected only from January 2014.

IQR, interquartile range.

At the first visit, 81.3% of women had blood pressure values consistent with the criteria for normal blood pressure (60.2%) or raised blood pressure (21.1%) by the American College of Cardiology-American Heart Association. We found that 15.5% of women had stage 1 hypertension and 3.1% had stage 2 hypertension, more than the 1.3% who reported having a history of chronic hypertension (table 1).

Based on traditional clinical risk factor screening for the risk of pre-eclampsia used in clinical care, 2.2% of women took aspirin for prevention of pre-eclampsia during pregnancy (table 1). On average, 94.4% of pregnancies ended at term, with 5.6% ending preterm, usually spontaneously. Pre-eclampsia developed in 2.9% of births and in 2.1% at term. The mode of birth was caesarean section in 27.0% of pregnancies, with 13.4% having a birth weight <10th centile. Perinatal mortality was 3.8/1000 births, and 5.7% of babies required admission to the neonatal intensive care unit.

In general, risk calibration was good (online supplemental figure S1a–e). Predicted incidences of term pre-eclampsia tended to be slightly overestimated because the Fetal Medicine Foundation model assumes delivery from no other cause. This generally good risk calibration supports the use of estimated risks to predict overall incidence. Tables2^5^ shows the expected reduction in pre-eclampsia associated with lowering diastolic blood pressure from 11-13 weeks’ gestation.

**Table 2:** Expected reduction in number of patients with pre-eclampsia in pregnancies screened for pre-eclampsia at 11-13 weeks' gestation, after treatment of diastolic blood pressure of >90 mm Hg to a target level of 85 mm Hg

Pre-eclampsia type*	All pregnancies (n=54 422)				Pregnancies with diastolic blood pressure >90 mm Hg (n=859, 1.58%)			
	Observed†	Expected‡	No (%)§	Relative risk reduction (%)	Observed†	Expected‡	No (%)§	Relative risk reduction (%)*
Preterm (<37 weeks)	453 (0.83)	482 (0.89)	455 (0.84)	5.65	77 (8.96)	69 (8.02)	42 (4.85)	39.50
<32 weeks	107 (0.20)	85 (0.16)	77 (0.14)	9.24	18 (2.10)	17 (1.93)	9 (1.02)	47.08
<34 weeks	198 (0.36)	175 (0.32)	161 (0.30)	7.86	37 (4.31)	31 (3.62)	17 (2.02)	44.23
Term (≥37 weeks)	1140 (2.09)	1467 (2.69)	1427 (2.62)	2.73	97 (11.29)	128 (14.9)	88 (10.24)	31.25
All pre-eclampsia	1593 (2.93)	1864 (3.43)	1809 (3.32)	2.94	174 (20.26)	173 (20.11)	118 (13.73)	31.72

Values are number (%) unless indicated otherwise.

Current clinical diastolic blood pressure target is 90 mm Hg.

* Relative risk reduction values for preterm, term, and all pre-eclampsia are based on a comparison between the modelled pre-eclampsia event rates under antihypertensive treatment and the observed pre-eclampsia event rates.

† Number of patients observed in the dataset of 54 422 records.

‡ Number of patients expected based on pre-eclampsia risk from the Fetal Medicine Foundation competing risks model at 11-13 weeks' gestation. Online supplemental figure S1a–eFigures S1a-e shows calibration plots between observed and expected risk of pre-eclampsia. In general, risk calibration was good, which supports use of estimated risks to predict overall incidence. Predicted incidences of term pre-eclampsia tended to be slightly overestimated because the Fetal Medicine Foundation model assumes delivery from no other cause.

§ Number of patients expected after treatment based on risk reduction associated with treatment of blood pressure, as specified.

**Table 3:** Expected reduction in number of patients with pre-eclampsia in pregnancies screened for pre-eclampsia at 11-13 weeks' gestation after treatment of diastolic blood pressure of >85 mm Hg to a target level of 85 mm Hg

Pre-eclampsia type*	All pregnancies (n=54 422)				Pregnancies with diastolic blood pressure>85 mm Hg (n=2611, 4.80%)			
	Observed†	Expected‡	No (%)§	Relative risk reduction (%)	Observed†	Expected‡	No (%)§	Relative risk reduction (%)*
Preterm (<37 weeks)	453 (0.83)	482 (0.89)	446 (0.82)	7.44	136 (5.21)	126 (4.84)	91 (3.47)	28.34
<32 weeks	107 (0.20)	85 (0.16)	75 (0.14)	11.69	37 (1.42)	29 (1.09)	19 (0.72)	34.61
<34 weeks	198 (0.36)	175 (0.32)	157 (0.29)	10.08	70 (2.68)	54 (2.08)	37 (1.41)	32.36
Term (≥37 weeks)	1140 (2.09)	1467 (2.69)	1412 (2.59)	3.75	225 (8.62)	263 (10.07)	208 (7.96)	20.91
All pre-eclampsia	1593 (2.93)	1864 (3.43)	1789 (3.29)	4.04	361 (13.83)	351 (13.45)	276 (10.56)	21.44

Values are number (%) unless indicated otherwise.

Current clinical diastolic blood pressure target is 90mm Hg

* Relative risk reduction values for preterm, term, and all pre-eclampsia are based on a comparison between the modelled pre-eclampsia event rates under antihypertensive treatment and the observed pre-eclampsia event rates.

† Number of patients observed in the dataset of 54422 records.

‡ Number of patients expected based on pre-eclampsia risk from the Fetal Medicine Foundation competing risks model at 11-13 weeks' gestation. Online supplemental figure S1a–e shows calibration plots between observed and expected risk of pre-eclampsia. In general, risk calibration was good, which supports use of estimated risks to predict overall incidence. Predicted incidences of term pre-eclampsia tended to be slightly overestimated because the Fetal Medicine Foundation model assumes delivery from no other cause.

§ Number of patients expected after treatment based on risk reduction associated with treatment of blood pressure, as specified.

**Table 4:** Expected reduction in number of patients with pre-eclampsia in pregnancies screened for pre-eclampsia at 11-13 weeks' gestation after treatment of diastolic blood pressure of >80 mm Hg to a target level of 80 mm Hg

Pre-eclampsia type*	All pregnancies (n=54 422)				Pregnancies with diastolic blood pressure>80 mm Hg (n=7202, 13.23%)			
	Observed†	Expected‡	No (%)§	Relative risk reduction (%)	Observed†	Expected‡	No (%)§	Relative risk reduction (%)*
Preterm (<37 weeks)	453 (0.83)	482 (0.89)	411 (0.75)	14.74	211 (2.93)	210 (2.92)	139 (1.93)	33.78
<32 weeks	107 (0.20)	85 (0.16)	67 (0.12)	21.05	54 (0.75)	44 (0.62)	27 (0.37)	40.12
<34 weeks	198 (0.36)	175 (0.32)	142 (0.26)	18.70	105 (1.46)	86 (1.19)	53 (0.74)	37.99
Term (≥37 weeks)	1140 (2.09)	1467 (2.69)	1342 (2.47)	8.46	428 (5.94)	495 (6.87)	371 (5.15)	25.08
All pre-eclampsia	1593 (2.93)	1864 (3.43)	1695 (3.11)	9.07	639 (8.87)	651 (9.04)	482 (6.69)	25.98

Values are number (%) unless indicated otherwise.

Current clinical diastolic blood pressure target is 90mm Hg.

* Relative risk reduction values for preterm, term, and all pre-eclampsia are based on a comparison between the modelled pre-eclampsia event rates under antihypertensive treatment and the observed pre-eclampsia event rates.

† Number of patients observed in the dataset of 54422 records.

‡ Number of patients expected based on pre-eclampsia risk from the Fetal Medicine Foundation competing risks model at 11-13 weeks' gestation. Online supplemental figure S1a–e shows calibration plots between observed and expected risk of pre-eclampsia. In general, risk calibration was good, which supports use of estimated risks to predict overall incidence. Predicted incidences of term pre-eclampsia tended to be slightly overestimated because the Fetal Medicine Foundation model assumes delivery from no other cause.

§ Number of patients expected after treatment based on risk reduction associated with treatment of blood pressure, as specified.

**Table 5:** Expected reduction in number of patients with pre-eclampsia in pregnancies screened for pre-eclampsia at 11-13 weeks' gestation after treatment of diastolic blood pressure of >75 mm Hg to a target level of 75 mm Hg

Pre-eclampsia type*	All pregnancies (n=54 422)				Pregnancies with diastolic blood pressure >75 mm Hg (n=16 049, 29.49%)			
	Observed*	Expected†	No (%)ǂ	Relative risk reduction (%)	Observed*	Expected†	No (%)ǂ	Relative risk reduction (%)*
Preterm (<37 weeks)	453 (0.83)	482 (0.89)	353 (0.65)	26.76	305 (1.90)	310 (1.93)	181 (1.13)	41.57
<32 weeks	107 (0.20)	85 (0.16)	55 (0.10)	34.41	72 (0.45)	61 (0.38)	32 (0.20)	47.70
<34 weeks	198 (0.36)	175 (0.32)	119 (0.22)	31.75	140 (0.87)	121 (0.75)	66 (0.41)	45.82
Term (≥37 weeks)	1140 (2.09)	1467 (2.69)	1213 (2.23)	17.32	678 (4.22)	806 (5.02)	552 (3.44)	31.51
All pre-eclampsia	1593 (2.93)	1864 (3.43)	1520 (2.79)	18.45	983 (6.12)	1047 (6.53)	704 (4.38)	32.83

Values are number (%) unless indicated otherwise.

Current clinical diastolic blood pressure target is 90mm Hg.

* Relative risk reduction values for preterm, term, and all pre-eclampsia are based on a comparison between the modelled pre-eclampsia event rates under antihypertensive treatment and the observed pre-eclampsia event rates.

† Number of patients observed in the dataset of 54422 records.

‡ Number of patients expected based on pre-eclampsia risk from the Fetal Medicine Foundation competing risks model at 11-13 weeks' gestation. Online supplemental figure S1a–e shows calibration plots between observed and expected risk of pre-eclampsia. In general, risk calibration was good, which supports use of estimated risks to predict overall incidence. Predicted incidences of term pre-eclampsia tended to be slightly overestimated because the Fetal Medicine Foundation model assumes delivery from no other cause.

§ Number of patients expected after treatment based on risk reduction associated with treatment of blood pressure, as specified.

The current standard of care is to treat a diastolic blood pressure threshold of >90 mm Hg to a target level of 85 mm Hg.^20^ Antihypertensive treatment given to the relevant 1.6% of the maternity population would be associated with a relative risk reduction of 31.7% in pre-eclampsia because of a slightly greater reduction in preterm pre-eclampsia. The number needed to benefit would be 15.6. For the maternity population overall, the relative risk reduction in pre-eclampsia would be 2.9%.

Decreasing the diastolic blood pressure threshold for antihypertensive treatment to >85 mm Hg and treating to a target level of diastolic blood pressure of 85 mm Hg would result in antihypertensive treatment being offered to 4.8% of the maternity population. This strategy would be associated with a relative risk reduction of 21.4% in pre-eclampsia because of a slightly greater reduction in preterm pre-eclampsia. The number needed to benefit would be 34.8. For the maternity population overall, the relative risk reduction in pre-eclampsia would be 4.0%.

Further decreasing the diastolic blood pressure threshold for antihypertensive treatment to >80 mm Hg and treating to a target level of diastolic blood pressure of 80 mm Hg would result in antihypertensive treatment being offered to 13.2% of the maternity population. This strategy would be associated with a relative risk reduction of 26.0% in pre-eclampsia because of a slightly greater reduction in preterm pre-eclampsia. The number needed to benefit would be 42.6. For the maternity population overall, the relative risk reduction in pre-eclampsia would be 9.1%.

Finally, decreasing the diastolic blood pressure threshold for antihypertensive treatment to >75 mm Hg and treating to a target level of diastolic blood pressure of 75 mm Hg would result in antihypertensive treatment being offered to 29.5% of the maternity population. This strategy would be associated with a 32.8% reduction in pre-eclampsia because of a greater reduction in preterm pre-eclampsia. The number needed to benefit would be 47.0. For the maternity population overall, the relative risk reduction in pre-eclampsia would be 18.5%.

## Discussion

### Principal findings

We have shown through modelling that from 11-13 weeks' gestation, antihypertensive treatment for diastolic blood pressure values of <90 mm Hg could reduce the incidence of pre-eclampsia by almost half, based on an ethnically and socioeconomically diverse cohort of >50 000 unselected pregnancies. Our analyses account for the risk of pre-eclampsia associated with established risk factors for pre-eclampsia (ie, maternal personal characteristics, past history, and current pregnancy characteristics), many of which also influence blood pressure levels, and all of which are accounted for in the Fetal Medicine Foundation competing risks model.

Although the effect on the maternity population overall would be small, unless women with a diastolic blood pressure of >75 mm Hg were offered antihypertensive treatment (modelled results showed that pre-eclampsia may be reduced by 18.4%), the number-needed-to-benefit for treatment of those with raised blood pressure would vary from 15.6 currently (for the 1.6% of the maternity population with a diastolic blood pressure of >90 mm Hg at 11-13 weeks’ gestation), to 35 (for treatment of the 4.8% of pregnancies with a diastolic blood pressure of >85 mm Hg), and up to 47 (for treatment of the 29.5% of the population with a diastolic blood pressure of >75 mm Hg). The beneficial effect would be on both preterm and term pre-eclampsia. Pre-eclampsia at term represents about 80% of the disease, which is responsible for more than half of maternal morbidity and a substantial minority of fetal or newborn morbidity.^34^

### Comparison with other studies

Systematic reviews have shown that although higher blood pressure in the first half of pregnancy is associated with more adverse pregnancy outcomes, only stage 2 hypertension (ie, blood pressure ≥140/90 mm Hg) has sufficiently good diagnostic test properties to distinguish between pregnancies at risk and not at risk of adverse outcomes.^8^ Using different thresholds for identifying an abnormal blood pressure, according to different maternal characteristics, might be beneficial, particularly for those who have underweight or for the approximately half of the maternity population who are parous with no history of pre-eclampsia.^10^ We found no individual patient data meta-analysis that explored the effects of these characteristics on the relation between blood pressure level and pre-eclampsia.

When antihypertensive treatment was first used in pregnancy, a reduction in the incidence of pre-eclampsia was expected. Although small individual trials were not successful in this regard, the effect of antihypertensive treatment of blood pressure measurements of ≥140/90 mm Hg on pre-eclampsia and other pregnancy outcomes has now been studied in large definitive randomised trials. The target diastolic blood pressure was 85 mm Hg in CHIPS^3^ and the target blood pressure was <140/90 mm Hg in the CHAP trial.^4^ These trials showed that breakthrough hypertension could still occur, but antihypertensive treatment halved the risk of progression to severe hypertension that might have otherwise developed. Also, this effect was associated with fewer maternal end organ complications believed to be related to endothelial dysfunction (eg, thrombocytopenia and raised levels of liver enzymes). Currently, however, the impact of lower blood pressure treatment thresholds and targets on outcomes in pregnancy has not been investigated. Although a secondary analysis of the CHAP trial cohort reported that those with lower average blood pressure levels (whether achieved spontaneously or after antihypertensive treatment) had a lower risk of adverse pregnancy outcomes,^35^ women were not randomised to these lower blood pressure targets.

Our findings suggest that when other risk factors for pre-eclampsia are accounted for, lowering the threshold for antihypertensive treatment from early pregnancy could have a clinically relevant effect on the overall incidence of pre-eclampsia at preterm and term gestational ages. Because women with chronic hypertension would be included in these groups and treated at lower thresholds of diastolic blood pressure, studying this intervention could be important because aspirin treatment is insufficient to reduce preterm pre-eclampsia in this particularly high risk group of women.^36^ Nevertheless, this blood pressure management strategy requires evaluation in a large definitive randomised controlled trial to examine the potential benefits, risks, and costs. Antihypertensive treatment is an inexpensive, well tolerated, and widely available intervention, and only a small percentage of women (about 2%) discontinue drug treatment because of maternal side effects. Also, the long held concern that antihypertensive drugs may impair fetal growth has been answered by reassuring definitive data from CHIPS^3^ and the CHAP^4^ trial.

### Strengths and limitations of this study

The strengths of our study include the large diverse cohort of unselected pregnancies, screened routinely for pre-eclampsia at 11-13 weeks' gestation by the most accurate method (competing risk model rather than clinical risk factors). Blood pressure measurements were standardised. We undertook prospective, detailed documentation of baseline characteristics (including self-identified ethnic group) and outcomes. The observed incidence of pre-eclampsia of 3% in our study cohort was consistent with rates in the general maternity population.

The major limitation of our analysis was that as a modelling exercise, we made several assumptions. Firstly, we assumed that reducing the calculated risk of pre-eclampsia (by reducing blood pressure) would likely directly affect the incidence of pre-eclampsia. Secondly, we also assumed that no apparent interaction exists between aspirin and the risk of pre-eclampsia, based on subgroup analyses of the ASPRE (Combined Multimarker Screening and Randomized Patient Treatment with Aspirin for Evidence-Based Pre-eclampsia Prevention) trial,^33^ and also no apparent decrease in the effect of aspirin by antihypertensive treatment because a statistically significant reduction in pre-eclampsia was seen in the CHAP trial where >75% of women in each group were receiving aspirin by the time of birth.^4^ Thirdly, our assumption of no effect of aspirin may have resulted in an overestimation of the benefits of antihypertensive treatment in reducing pre-eclampsia; overestimation of the effect of lowering blood pressure for the prevention of pre-eclampsia was likely to have been small (eg, 20.4% relative risk reduction, rather than 21.4% when treating those with a diastolic blood pressure of >85 mm Hg to a target level of 85 mm Hg), assuming identification of risk based on the Fetal Medicine Foundation competing risks model, acceptance of and adherence to aspirin by those offered this preventive treatment, and an approximate halving of the risk of preterm pre-eclampsia with aspirin (based on a detection rate of 75% of the competing risks model and a 62% relative risk reduction of pre-eclampsia with aspirin).^33^ Fourthly, whether diastolic blood pressure achieved with antihypertensive treatment would be associated with a similar risk of pre-eclampsia to that achieved spontaneously requires investigation in a prospective study, ideally a randomised trial of blood pressure targets.

Other limitations were that for all pre-eclampsia and term pre-eclampsia, we slightly overestimated the risk for birth with pre-eclampsia; although we could account for censoring for birth from other causes, this approach would add complexity while not adding to our stated aims. All participants had singleton pregnancies and so our results do not necessarily apply to twin or high order multiple pregnancies. Hypertension was treated in accordance with national guidance during the study period (mainly systolic blood pressure of ≥150 mm Hg or diastolic blood pressure of ≥100 mm Hg^26^) until June 2019, when the threshold for intervention was a systolic blood pressure of ≥140 mm Hg or diastolic blood pressure of ≥90 mm Hg.^20^ Although the relation between first trimester blood pressure threshold and pre-eclampsia may have been reduced by antihypertensive treatment in women with chronic hypertension or by starting treatment with aspirin for prevention of pre-eclampsia in women at increased risk, the proportion of women treated in this way was small (<4% overall). Finally, our data do not provide information on the cost consequences of antihypertensive treatment for a diastolic blood pressure of above 85 mm Hg or above lower thresholds, as a potential intervention. Also, although patients were not involved in the study design or conduct, a James Lind Alliance Blood Pressure in Pregnancy Priority Setting Partnership identified as a top 10 priority how best to manage hypertension in pregnancy with antihypertensive drugs.^37^

### Conclusions

Adjusting for factors that affect the risk of pre-eclampsia and blood pressure level, this modelling study suggested that lowering blood pressure from early pregnancy may reduce preterm and term pre-eclampsia by up to almost half in those treated. A large definitive intervention trial is needed to determine the benefits, risks, and costs of this blood pressure lowering strategy for clinical practice.

## Supplementary material

10.1136/bmjmed-2025-001631 online supplemental file 1

10.1136/bmjmed-2025-001631 online supplemental file 2

## Data Availability

Data are available upon reasonable request.
