# Supplementary material for: Modelling lowering of raised blood pressure in pregnancy to reduce pre-eclampsia: secondary analysis of data from prospective cohort studies
Source: BMJ Med. 2026 Mar 27;5(1):e001631. doi: 10.1136/bmjmed-2025-001631 (PMC13034301; doi:10.1136/bmjmed-2025-001631)

SUPPLEMENTARY APPENDIX

	Title	Page number
**Tables**		
S1	Model for simulation of systolic blood pressure from diastolic blood pressure	2
**Figures**		
S1	Calibration plots of observed incidence of PE against predicted incidence from pre-eclampsia risk	3
S1a	All PE	3
S2	Term PE	3
S3	Preterm PE <37 weeks	4
S4	Preterm PE <34 weeks	4
S5	Preterm PE <32 weeks	5

Table S1: Model for simulation of systolic blood pressure from diastolic blood pressure

	Estimate	Lower confidence limit	Upper confidence limit	p-value
Intercept	2.7552	2.5174	2.9930	<0.0001
log10(DBP)	-1.3304	-1.5873	-1.0736	<0.0001
log10(DBP)^2	0.5175	0.4481	0.5868	<0.0001

DBP (diastolic blood pressure)

Figure S1: Calibration plots of observed incidence of PE against predicted incidence from PE risk for all PE (a), term PE (b), pre-term PE (c), PE < 34 weeks (d) and PE < 32 weeks (e).

AUC (area under the receiver operating curve), MAP (mean arterial pressure), PE (pre-eclampsia), PLGF (placental growth factor), UTPI (uterine artery pulsatility index)

Legend: The solid grey line is the line of equality, which represents perfect calibration. The overall incidence is shown by the horizontal interrupted line, and the histograms show the distribution of risks in pregnancies with PE (red) and those without (grey). Calibration intercept and slope are presented alongside AUC.

Figure S1a: Calibration plot for **all PE**

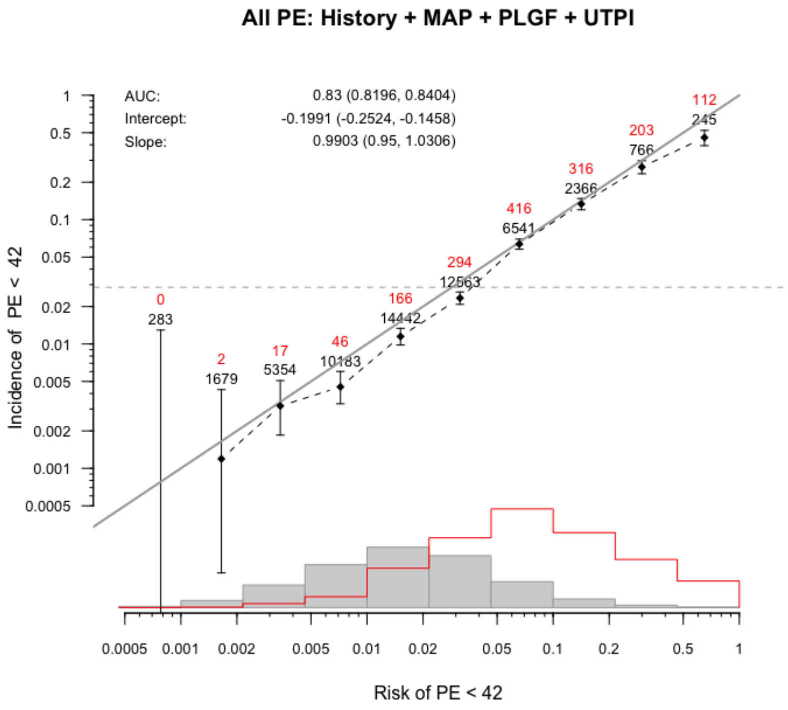

Figure S1b: Calibration plot for **term PE** (≥37 weeks)

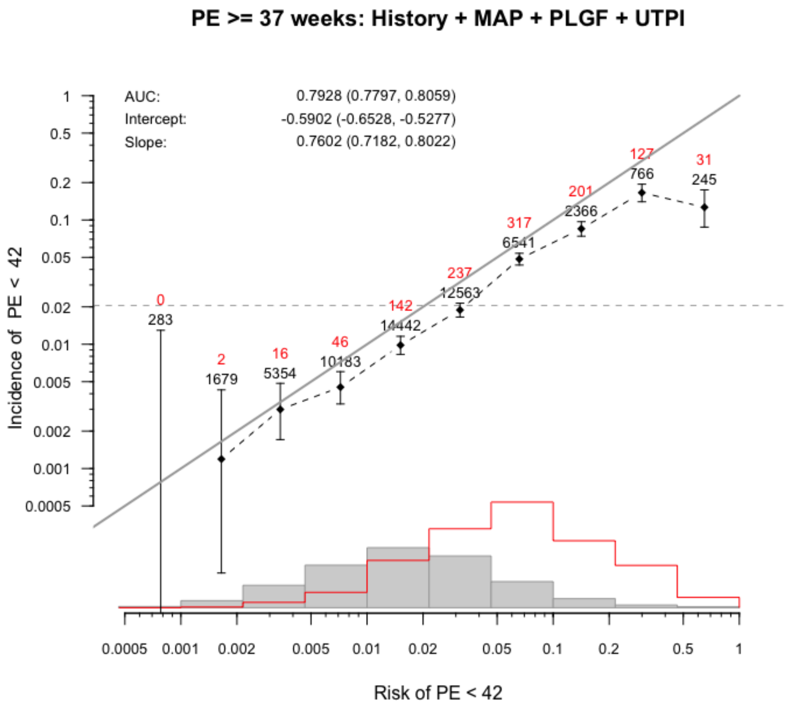

Figure S1c: Calibration plot for **preterm PE** (<37 weeks)

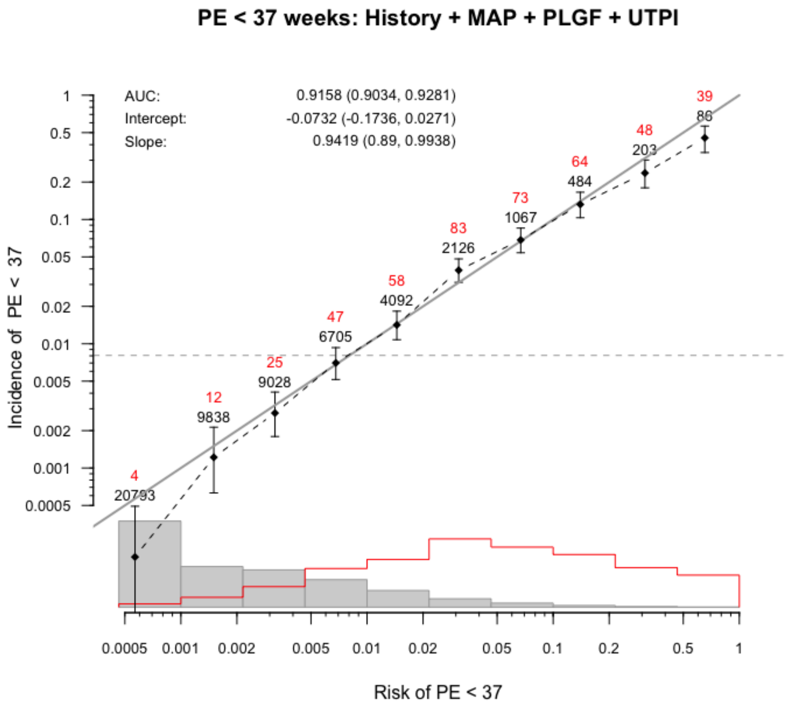

Figure S1d: Calibration plot for **preterm PE at <34 weeks**

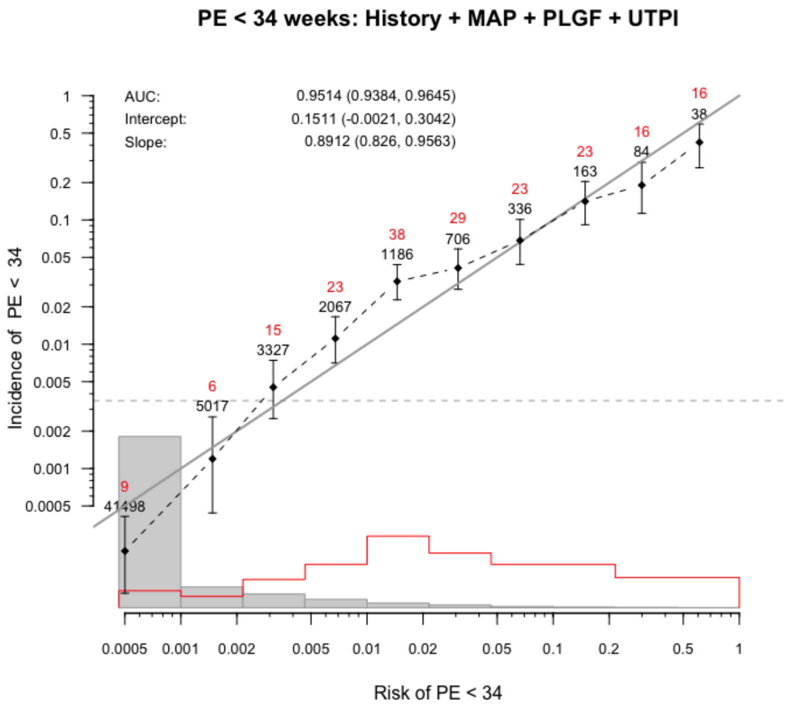

Figure S1e: Calibration plot for **preterm PE at <32 weeks**

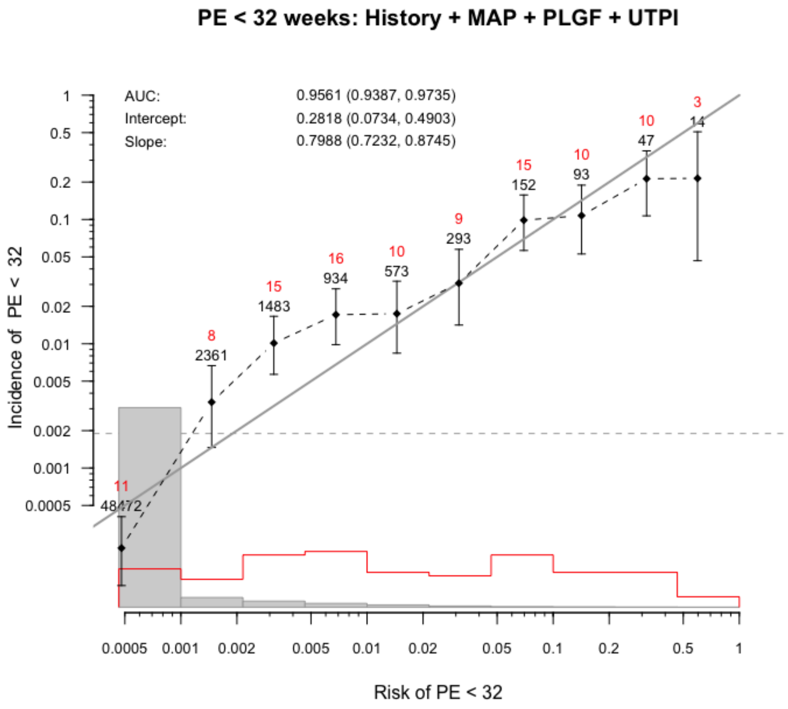

Supplement: online supplemental file 1 [file bmjmed-5-1-s001.pdf]
