# Supplementary material for: Modelling lowering of raised blood pressure in pregnancy to reduce pre-eclampsia: secondary analysis of data from prospective cohort studies
Source: BMJ Med. 2026 Mar 27;5(1):e001631. doi: 10.1136/bmjmed-2025-001631 (PMC13034301; doi:10.1136/bmjmed-2025-001631)
Supplement: Supplementary file 3 [file bmjmed-2025-001631.reviewer_comments.pdf]

PEER REVIEW HISTORY

BMJ Medicine publishes all reviews undertaken for accepted manuscripts. Reviewers are asked to complete a checklist review form and are provided with free text boxes to elaborate on their assessment. These free text comments are reproduced below.

ARTICLE DETAILS

**TITLE (PROVISIONAL)**	Modelling lowering of elevated blood pressure in pregnancy to reduce pre-eclampsia
**AUTHORS**	Magee, Laura; Wright, Alan; Syngelaki, Argyro; Wright, David; von Dadelszen, Peter; NICOLAIDES, Kypros

VERSION 1 - REVIEW

**REVIEWER NAME**	Pressman, Eva
**REVIEWER AFFILIATION**	University of Rochester, OB/Gyn
**REVIEWER CONFLICT OF INTEREST**	None
**AI DISCLOSURE**	No
**DATE REVIEW RETURNED**	12-May-2025

**GENERAL COMMENTS**	This is a large study that models the potential effects of initiating antihypertensive therapy at lower diastolic pressures to achieve lower diastolic BP thresholds. Overall the information provided is useful and important but as noted in the discussion of the limitations, modelling cannot accurately predict actual outcomes in many cases. Two additional issues need attention. The first is aspirin use which is addressed but not fully. It would seem that given the data that aspirin can decrease the risk and complications from preeclampsia in many pregnancies, prescribing aspirin in addition to antihypertensives in this model would make sense and should be evaluated more fully. Most importantly, this model does not include any potential risks from expanding the use of antihypertensive medications to almost 30% of pregnant patients. There should be at least a mention of potential financial implications, use of additional testing such as ultrasounds or NSTs for patients on antihypertensive medications and impacts on fetal growth.

**REVIEWER NAME**	Ganzevoort, Wessel
**REVIEWER AFFILIATION**	Amsterdam UMC University of Amsterdam, Obstetrics and Gynecology
**REVIEWER CONFLICT OF INTEREST**	I know authors Magee and von Dadelszen pretty well personally and professionally, we have published a couple of times. However, I think I can give an unbiased review.
**AI DISCLOSURE**	No
**DATE REVIEW RETURNED**	30-May-2025

**GENERAL COMMENTS**	With interest I read this manuscript. It is methodologically excellent and a good addition to literature.<br><br>I think I have one major concern of a topic that needs better addressing, although it has been discussed somewhat in the

	paragraph on page 11 290-299:<br>The authors have modeled that lowering blood pressure leads to fewer cases of pre-eclampsia. This appears to be self-evident since high blood pressure is one of the pillars of the diagnoses of pre-eclampsia. So this is an unsurprising finding. I would like the authors to discuss more in-depth the relationship between blood pressure achieved after antihypertensive medication and the endothelial dysfunction? Is antihypertensive medication actually more than symptomatic treatment, is it improving endothelial dysfunction? And from that, does it change the risk of the adverse outcomes that potentially affect the risk of severe morbidities? Or is it just changing the measurement of 'blood pressure' and does the underlying risk resulting from endothelial dysfunction remain? In case of the first, what is an estimated NNT to prevent any severe morbidity? This is likely high given that the real adverse outcomes that everybody wishes to prevent, i.e. the ones that immediately jeopardize life, are 2.4% in CHAP and 2.8% in CHIPS, with CI including 1.0. In case of the second, the case can be made that we protect the patient from unnecessary iatrogenic premature births triggered by the diagnosis of pre-eclampsia? [which may be a benefit in itself]<br>The CHIPS and CHAP have shown<br>I think the uncertainty of first or second needs to be addressed, as well as the 'what we are preventing'. This is a little philosophical, but it is important because it is the human behavior among doctors that sometimes really averts adverse outcomes and sometimes is just risk averse without actual benefits or sometimes even harm.<br><br>Minor:<br>the sentence on page 8 4th line "this means that women with chronic...." is incomplete?<br>Typo page 10 line 256 'toa' and line 266 'based an'

**REVIEWER NAME**	Perera, Rafael
**REVIEWER AFFILIATION**	University of Oxford, Primary Care Health Sciences
**REVIEWER CONFLICT OF INTEREST**	None
**AI DISCLOSURE**	I have used Grammarly to help with spelling and grammar, making the Review easier to read.
**DATE REVIEW RETURNED**	01-Jul-2025

**GENERAL COMMENTS**	bmjmed-2025-001631: Modelling lowering of elevated blood pressure in pregnancy to reduce pre-eclampsia<br><br>Stats Review<br><br>In this manuscript, the authors present results from a modelling study using the FMF model and data from three prospective screening studies for Pre-eclampsia (PE) in women attending maternity hospitals in England, Spain, Belgium, Italy and Greece, to evaluate the impact of a change in the threshold for treatment, based on diastolic blood pressure (dBP), with antihypertensives would have on the proportion of women with preterm and term PE. They find that a reduction in the threshold, which would therefore include more women in the treatment group, would reduce preterm and term PE. They use this to call for a large trial to evaluate this theoretical result. Although this is a useful next step, several key points need to be addressed and considered before these results can be used to support a large-scale trial.

	Major points:<br><br>The study relies on the FMF model as the engine to drive the results. A detailed introduction to this model is necessary, including its creation, purpose, and validation. In particular, the authors need to demonstrate the FMF validity of the data used in the study.<br><br>For this, a more comprehensive description of the screening studies, including details about each study, is required in the methods or supplementary material. In particular, it would be useful to understand national and study variation and to define the generalisability of the FMF model to these data. This is partly because Table 2 highlights that the model is not well-calibrated to the entirety of these results.<br><br>A major point is that the modelling results are based on assumptions about the impact that antihypertensives will have on dBP and how this translates to a reduction in PE. Multiple antihypertensives are contraindicated in pregnancy, so clarification of which ones are proposed, with an estimation of their potential effect on dBP, and ideally a clear causal estimate of the effect of this on reducing preterm and term PE. If possible, these could be obtained from previous evidence or an analysis of the data that the authors have, which might provide some estimate of this. They could conduct a natural experiment by examining the date when the change in the treatment threshold occurred (June 2019), or by studying women at the threshold for treatment (just before and just after) and estimating the potential effect on both dBP and PE.<br><br>Another major assumption which is not considered is that the modelling assumes a lack of adverse effects by the intervention, which is likely a major limitation of providing treatment to ever-decreasing threshold levels (and a higher number of women). If the FMF model treats BP as a continuous measure of risk, it is not surprising that a reduction of any kind would lead to an expected reduction in PE. The critical point here is that any benefit in BP reduction will need to be balanced with potential harms. These could be treatment-specific (drug-dependent) or more general.<br><br>In the description of the information included in the FMF model, the sBP was estimated based on dBP. Was this because sBP was not available? It is unclear why it would have to be estimated. Related to this, treatment with antihypertensives would have an average estimated impact on dBP and sBP, which could then be used to populate the model. Why was this not done this way?<br><br>In general, for all the assumptions made, please provide as much justification as possible, either through direct evidence or from previous literature.<br><br>Table 2: The model appears to be poorly calibrated. Some exploration of this might help. More information in the label about what each row means would be helpful. For example, are these PE at delivery or PE at any time (for ALL PE)? Some explanation as to why they do not add up (to ALL PE) is needed. Also, information about the independence of the rows (or lack thereof) is needed here. Do individuals appear on multiple rows, or are these mutually exclusive? Another point, the RRR estimates are close to a simple direct comparison (assuming the same denominator for Expected

	under the FMF and under the specific treatment regime). What approach was used to determine RRR? Please provide this in the Methods section.<br><br>Figure 1 (a and b): These Figures are difficult to compare because they use different scales. Visually, they appear to be the same graph. A more helpful approach would be to keep the same scale for both, which would allow a more precise visual comparison of the different risk levels.<br><br>Minor:<br><br>Typo (word missing) in<br>“ This means that women with chronic hypertension would be more often if their dBP were >85mmHg”<br><br>The Tweetable comment is overstating the findings. This study is not able to say anything directly about the causal association between medication to lower BP and PE.

VERSION 1 – AUTHOR RESPONSE

Reviewer: 1 (Dr. Eva Pressman, University of Rochester)

Comments to the Author

This is a large study that models the potential effects of initiating antihypertensive therapy at lower diastolic pressures to achieve lower diastolic BP thresholds. Overall the information provided is useful and important but as noted in the discussion of the limitations, modelling cannot accurately predict actual outcomes in many cases.

Response: Thank you for the positive comments. We trust that our conclusion makes clear that a large, definitive intervention trial would be needed to inform practice.

Two additional issues need attention. The first is aspirin use which is addressed but not fully. It would seem that given the data that aspirin can decrease the risk and complications from preeclampsia in many pregnancies, prescribing aspirin in addition to antihypertensives in this model would make sense and should be evaluated more fully.

Response: Thank you for giving us the opportunity to address this issue. Our challenge is that we do not feel that we can accurately predict the effect of aspirin use on PE incidence. We would like to take this opportunity to better explain our rationale.

- **First, aspirin administration in this study cohort was based on clinical risk factors (as per clinical guidance at the time), which have a 40% detection rate for preterm PE, for which aspirin is administered. (Aspirin does not decrease the risk of term PE, which represents the majority of all PE.)**
- **Aspirin is underutilised when prescribed according to clinical risk factors. For example, only 2.2% of the cohort took aspirin, but 3.1% were parous with previous PE, a major and well-recognised risk factor.**
- **Aspirin is highly effective in preventing preterm PE (by 60%) when risk is identified by the competing risks, multivariable model, and administered at a dose of 150mg/night, according to the findings of the ASPRE trial. While that is not how aspirin was administered in the study cohort, in our limitations, we did provide an estimate if aspirin had been administered as per ASPRE; this provided an estimate of by how much we may have overestimated the effect of BP modification on reducing the risk of all PE. The fact that the impact was estimated to be small reflects that only a maximum of 25% of PE occurs preterm, so the incidence of PE is driven primarily by the incidence of PE at term gestational age.**

As stated in the limitations, there is no apparent interaction between aspirin and PE risk, based on subgroup analyses of the ASPRE trial, and no apparent attenuation of aspirin effect

by antihypertensive therapy, based on the results of the CHAP trial. In that RCT, a significant reduction in PE was observed despite just over 75% of women in each group taking aspirin.

Finally, we should clarify that aspirin does not decrease complications of PE, but rather, the diagnosis whether defined traditionally (as hypertension and new-onset proteinuria in the second half of pregnancy) or broadly (as hypertension and either new-onset proteinuria, other maternal end-organ dysfunction, or uteroplacental dysfunction).

Most importantly, this model does not include any potential risks from expanding the use of antihypertensive medications to almost 30% of pregnant patients. There should be at least a mention of potential financial implications, use of additional testing such as ultrasounds or NSTs for patients on antihypertensive medications and impacts on fetal growth.

Response: Thank you for raising this excellent point. We have revised our Research Implications section, as follows: "Our findings suggest that once other risk factors for PE are accounted for, lowering of elevated BP values (and not just those of at least 140mmHg systolic and/or 90mmHg diastolic) may reduce the incidence of PE. This would require evaluation in a large, definitive randomised controlled trial, to examine these potential benefits and potential risks. While antihypertensive therapy is an inexpensive, well-tolerated, and widely-available intervention, globally, a small percentage of women (about 2%) discontinue medication due to maternal side effects; the long-held concern that antihypertensives may impair fetal growth has been addressed by reassuring, definitive data from the CHIPS(3) and CHAP(4) trials" (p13).

Reviewer: 2 (Dr. Wessel Ganzevoort, Amsterdam UMC University of Amsterdam)

Comments to the Author

With interest I read this manuscript. It is methodologically excellent and a good addition to literature.

I think I have one major concern of a topic that needs better addressing, although it has been discussed somewhat in the paragraph on page 11 290-299: The authors have modeled that lowering blood pressure leads to fewer cases of pre-eclampsia. This appears to be self-evident since high blood pressure is one of the pillars of the diagnoses of pre-eclampsia. So this is an unsurprising finding. I would like the authors to discuss more in-depth the relationship between blood pressure achieved after antihypertensive medication and the endothelial dysfunction? Is antihypertensive medication actually more than symptomatic treatment, is it improving endothelial dysfunction? And from that, does it change the risk of the adverse outcomes that potentially affect the risk of severe morbidities? Or is it just changing the measurement of 'blood pressure' and does the underlying risk resulting from endothelial dysfunction remain?

Response: Antihypertensive therapy was originally proposed to reduce the incidence of PE. While small individual trials were not able to demonstrate success in this regard, the adequately-powered CHIPS and CHAP trials were. Not only was antihypertensive therapy able to halve the risk of progression to severe hypertension that might have otherwise developed, but therapy was able to reduce associated end-organ complications (e.g., thrombocytopaenia and elevated liver enzymes) that the medical community believes reflect endothelial dysfunction.

In case of the first, what is an estimated NNT to prevent any severe morbidity? This is likely high given that the real adverse outcomes that everybody wishes to prevent, i.e. the ones that immediately jeopardize life, are 2.4% in CHAP and 2.8% in CHIPS, with CI including 1.0. In case of the second, the case can be made that we protect the patient from unnecessary iatrogenic premature births triggered by the diagnosis of pre-eclampsia? [which may be a benefit in itself] The CHIPS and CHAP have shown I think the uncertainty of first or second needs to be addressed, as well as the 'what we are preventing'. This is a little philosophical, but it is important because it is the human behavior among doctors that sometimes really averts adverse outcomes and sometimes is just risk averse without actual benefits or sometimes even harm.

Response: Thank you for these insightful comments. While prevention of serious maternal complications is the ultimate goal for the mother, alongside prevention of fetal/newborn complications, we have validated surrogates for those complications in severe hypertension and the diagnosis of PE, particularly as there were no demonstrable harms of BP lowering for the baby. An individual participant meta-analysis would further delineate whether there is any

measurable impact of antihypertensive therapy on serious maternal complications, and this has been offered by the CHIPS investigators to the CHAP team. In the meantime, if one were to consider the incidence of serious maternal complications with BP normalisation (vs. control) in CHIPS (2.0% vs. 3.7%) and CHAP (2.1% vs. 2.8%), the NNT to benefit would be 59 to 143. These are not dissimilar to the NNT to prevent eclampsia with MgSO4 (i.e., 50 for non-severe PE and 100 for severe PE), with an intervention (antihypertensive therapy) that is less costly (than MgSO4, due in well-resourced settings to the health professional costs).

Minor:

the sentence on page 8 4th line "this means that women with chronic...." is incomplete?

Response: You are quite right! We apologise for this error, and have revised the sentence, as follows: "This means that if their dBP were >85mmHg (whether or not on antihypertensive therapy), more women with chronic hypertension would be treated with antihypertensive and those on treatment would receive more medication, as their target BP would be lower (80 or 75mmHg) than at the present" (p9).

Typo page 10 line 256 'toa' and line 266 'based an'

Response: We apologise for these errors, and have revised "toa" to "to a" and "based an" to "based on" (p9).

Reviewer: 3 (Prof. Rafael Perera, University of Oxford)

Comments to the Author

Stats Review

In this manuscript, the authors present results from a modelling study using the FMF model and data from three prospective screening studies for Pre-eclampsia (PE) in women attending maternity hospitals in England, Spain, Belgium, Italy and Greece, to evaluate the impact of a change in the threshold for treatment, based on diastolic blood pressure (dBP), with antihypertensives would have on the proportion of women with preterm and term PE. They find that a reduction in the threshold, which would therefore include more women in the treatment group, would reduce preterm and term PE. They use this to call for a large trial to evaluate this theoretical result. Although this is a useful next step, several key points need to be addressed and considered before these results can be used to support a large-scale trial.

Response: Thank you for your detailed comments. We believe that we have addressed your concerns, but we would be happy to provide further clarification or make further revisions, as the reviewer sees fit.

Major points:

The study relies on the FMF model as the engine to drive the results. A detailed introduction to this model is necessary, including its creation, purpose, and validation. In particular, the authors need to demonstrate the FMF validity of the data used in the study.

For this, a more comprehensive description of the screening studies, including details about each study, is required in the methods or supplementary material. In particular, it would be useful to understand national and study variation and to define the generalisability of the FMF model to these data. This is partly because Table 2 highlights that the model is not well-calibrated to the entirety of these results.

Response: This is a very valid point, and appreciate the feedback. We have now added an explanatory paragraph to the Methods, as follows: "The Fetal Medicine Foundation (FMF) multivariable competing-risks model was applied for assessment of PE risk at 11-13 weeks' gestation. This model incorporates maternal factors and biomarkers, and is based on a survival-time model for the gestational age at delivery with PE(12). It is assumed that if the pregnancy were to continue indefinitely, all women would develop PE and whether they do so or not before a specific gestational age depends on competition between birth before or after PE develops. A Gaussian model for gestational age at delivery was chosen, based on model fit and interpretability. In pregnancies at low risk of PE, maternal factors and biomarkers serve to shift the gestational age distribution to the right, meaning that most births will occur before PE

develops. In pregnancies at high risk of PE, maternal factors and biomarkers serve to shift the gestational age distribution to the left, so the smaller the mean gestational age, the higher is the risk for PE. This means that PE risk-scoring is continuous and the screen-positive rate (SPR) can be adjusted to the desired population risk cut-off. The model has been externally validated in a mixed UK and European population(13), as well as those in Asia(14), Spain(15), and Denmark(16), and is available online at 'FetalMedicine.org'" (p6).

A major point is that the modelling results are based on assumptions about the impact that antihypertensives will have on dBP and how this translates to a reduction in PE. Multiple antihypertensives are contraindicated in pregnancy, so clarification of which ones are proposed, with an estimation of their potential effect on dBP, and ideally a clear causal estimate of the effect of this on reducing preterm and term PE. If possible, these could be obtained from previous evidence or an analysis of the data that the authors have, which might provide some estimate of this. They could conduct a natural experiment by examining the date when the change in the treatment threshold occurred (June 2019), or by studying women at the threshold for treatment (just before and just after) and estimating the potential effect on both dBP and PE.

Response: Thank you for pointing out the important omission of critical information. In the Methods, we have now added an 'Antihypertensive therapy' section, above 'Outcomes'. This new section details the current approach to antihypertensive therapy, the contemporaneous approach during the time when participants were enrolled (with the reference showing by country, that BP control was not advocated in the countries where participants were enrolled - England, Spain, Belgium, Italy, Greece), choice of antihypertensive agents, their impact on BP and PE, and the lack of influence of antihypertensives on fetal growth restriction, a long-standing concern about antihypertensive therapy. The addition is as below. Also, in our Analysis section (p9), we have specified that our assumptions are based on use of any of the commonly-used antihypertensive agents in pregnancy.

"*Antihypertensive therapy*

The current BP treatment approach is to offer antihypertensive medication to women with dBP ≥90mmHg, to achieve a target dBP of 85mmg(20), based on the results of the CHIPS and CHAP trials. However, as these trials were published in 2015 and 2022, respectively, BP control below 140/90mmHg in pregnancy has been advocated only in the last 5-10 years(21).

We did not restrict our interest to use of any specific antihypertensive agent. While labetalol is by far, the antihypertensive agent of choice in pregnancy for non-severe hypertension, there is a wide range of inexpensive, widely-available options. (Only RAAS [renin-angiotensin-aldosterone system] blockers contraindicated for use in pregnancy, based on their fetotoxicity.) In a network meta-analysis of 61 informative randomised trials, all commonly-prescribed antihypertensives (labetalol, other β-blockers, methyldopa, calcium channel blockers, and mixed/multi-drug therapy) versus placebo/no therapy halved the risk of severe hypertension (range: 30% to 70%), but there were few clear differences between them, and 95% credible intervals were wide. Labetalol decreased proteinuria (presumably of PE) (odds ratio [OR] 0.73 [95% credible interval, 0.54–0.99]) and fetal/newborn death (OR 0.54 [0.30–0.98]) compared with placebo/no therapy, and proteinuria compared with methyldopa (OR 0.66 [0.44–0.99]) and calcium channel blockers (OR 0.63 [0.41–0.96]). Also, antihypertensives in pregnancy are associated with a very low rate of adverse effects (<5%), as no difference has been demonstrated between antihypertensives and placebo/no therapy or other antihypertensives, albeit with wide 95% confidence intervals(22, 23)

Antihypertensive therapy in pregnancy lowers BP, with the magnitude of effect dependent on the target BP. The goal of lowering diastolic BP by 5mmHg, to a target diastolic BP of 85mmgHg, was achieved in the CHIPS trial(3). Lowering of BP of this magnitude was sufficient to favourably impact maternal outcomes (of severe hypertension and severe features of PE), and importantly, without having a negative impact on fetal growth. BP was lowered (systolic/diastolic) by 5.8/4.6mmHg in CHIPS, and 3.1/2.4mmHg in CHAP. The severe features of PE were 38% of control in CHIPS (based on platelet count <100x109/L) and 80% in CHAP. This suggests that for a roughly double the BP reduction in CHIPS, one achieved a halving of the incidence of severe features of PE" (p7).

Another major assumption which is not considered is that the modelling assumes a lack of adverse effects by the intervention, which is likely a major limitation of providing treatment to ever-decreasing threshold levels (and a higher number of women). If the FMF model treats BP as a continuous

measure of risk, it is not surprising that a reduction of any kind would lead to an expected reduction in PE. The critical point here is that any benefit in BP reduction will need to be balanced with potential harms. These could be treatment-specific (drug-dependent) or more general.

Response: Thank you again for pointing out this omission. We have added side effects to our assumptions, as follows: "Furthermore, we assumed that greater falls in BP would achieve greater falls in PE, and that adverse effects would be negligible (see 'Antihypertensive therapy', above)" (p10), and "Also, antihypertensives in pregnancy are associated with a very low rate of adverse effects (≈2%), as no difference has been demonstrated between antihypertensives and placebo/no therapy or other antihypertensives, albeit with wide 95% confidence intervals(22, 23)" (p7).

Also, we have revised our Discussion, to include the issue of 'breakthrough' hypertension and the concern that prevention of PE might be because the diagnostic criterion of hypertension is being prevented, as follows: "When first used in pregnancy, it was hoped that antihypertensive therapy would reduce the incidence of PE. While small individual trials were not able to demonstrate success in this regard, the impact of antihypertensive treatment of BP ≥140/90 mmHg on PE and other pregnancy outcomes has now been studied in large, definitive randomised trials. The target dBP was 85 mmHg in the Control of Hypertension In Pregnancy Study (CHIPS) trial(3), and the target BP was <140/90 mmHg in the Chronic Hypertension And Pregnancy (CHAP) trial(4). Importantly, these trials showed that 'breakthrough hypertension' could still occur, but antihypertensive therapy halved the risk of progression to severe hypertension that might have otherwise developed, and this was associated with fewer maternal end-organ complications believed to be related to endothelial dysfunction (e.g., thrombocytopaenia and elevated liver enzymes)" (p13).

In the description of the information included in the FMF model, the sBP was estimated based on dBP. Was this because sBP was not available? It is unclear why it would have to be estimated. Related to this, treatment with antihypertensives would have an average estimated impact on dBP and sBP, which could then be used to populate the model. Why was this not done this way?

Response: First, all participants had systolic and diastolic BP values available, that went into the calculation of their PE risk.

Second, in the modelling exercise, we focussed our attention on diastolic BP, for ease, as had been done in the CHIPS trial. As we were modelling the potential impact of lowering diastolic BP with antihypertensive therapy, we simulated the associated systolic BP; we have added further explanation about this in the Methods: "In our approach, dBP values exceeding a specified threshold were fixed at that threshold, and systolic BP values were simulated using a predefined model (Table S1), under the assumption that a reduction in dBP would correspond to a reduction in systolic BP. Mean arterial pressure and the risk of delivery with PE were then recalculated, and these updated risk estimates were used to model PE incidence" (p9).

Third, how much antihypertensive therapy lowers BP is based on one's target, akin to adjusting a thermostat to achieve a target room temperature. What we can say, is that in the CHIPS trial, a target drop in BP of 5mmHg (based on the baseline BP in the 'tight' control group, and the diastolic BP goal of 85mmHg), was achieved. Therefore, we are assuming that we can achieve other target diastolic BP values, of 80 and 75mmHg in the analysis that is the subject of the submitted manuscript.

In general, for all the assumptions made, please provide as much justification as possible, either through direct evidence or from previous literature.

Response: Thank you for your steer on this. As per the responses above, we have added to our background on antihypertensive therapy (p7), leading to further justification for our assumptions (p10). We are very happy to provide further detail and justification, as required.

Table 2: The model appears to be poorly calibrated. Some exploration of this might help. More information in the label about what each row means would be helpful. For example, are these PE at delivery or PE at any time (for ALL PE)? Some explanation as to why they do not add up (to ALL PE) is needed. Also, information about the independence of the rows (or lack thereof) is needed here. Do individuals appear on multiple rows, or are these mutually exclusive?

Response: Thank you for the opportunity to clarify the issue of calibration and the data presented in Table 2.

Thank you for this observation about the model calibration. We agree that some of these numbers indicate less that desirable risk calibration, particularly for PE<32 and PE < 34 weeks. These cases of early and very early PE are rare, therefore there is considerable uncertainty in risk estimation and calibration. For term and all PE, we slightly overestimate risk for delivery with PE because the model assumes delivery from no other cause. While we could account for censoring in these numbers, we feel that this would add complexity without adding to the aims of the paper.

As part of our response, we have produced calibration plots, where we plot observed incidence against that predicted from risk by grouping data into bins according to risk (Figures S1a to S1e, for all PE, term PE, preterm PE, and preterm PE <34 or <32 weeks). We quantified calibration by the estimated intercept from a logistic regression of incidence on the logit of risk with the slope fixed at 1 and by the estimated slope of the regression line of the logistic regression of incidence on the logit of risk. For perfectly calibrated risks, the intercept should and the slope should be 1.0. As can be seen, overall, these plots demonstrate good calibration.

These points have been added to the paper in the following places:

- **Methods: "Model calibration was checked by calibration plots, in which observed PE incidence was plotted against predicted PE incidence from risk, by grouping data into bins according to PE risk. We quantified calibration by the estimated intercept from a logistic regression of PE incidence on the logit of PE risk, with the slope fixed at 1, and by the estimated slope of the regression line of the logistic regression of incidence on the logit of PE risk. For perfectly calibrated risks, the intercept should be 0 and slope should be 1.0", p9),**
- **Results: "In general, risk calibration was good, as illustrated in Figures S1 a-e. Predicted incidences of term PE tended to be slightly overestimated, as the FMF model assumes delivery from no other cause. This generally good risk calibration supports the use of estimated risks to predict overall incidence" (p11); and**
- **Discussion/limitations: "Additional limitations included that for all PE and term PE, we slightly overestimated risk for birth with PE; while we could account for censoring for birth from other causes, this would add complexity whilst not adding our stated aims" (p14).**

Second, we have clarified Table 2 by adding an explanatory footnote for PE type, making it clearer that preterm PE is presented overall, and then by subgroups of <32 and <34 weeks. We have added a specific row for term PE, and made it clearer that preterm and term PE add to all PE. We believe that this now explains the independence of the rows, but we are happy to clarify further, as needed. Also, we noted that the 95% CI for the RRR were missing, for which we apologise; these have now been added.

Table 2: Another point, the RRR estimates are close to a simple direct comparison (assuming the same denominator for Expected under the FMF and under the specific treatment regime). What approach was used to determine RRR? Please provide this in the Methods section.

Response: We have revised our Methods to include further explanation of the RRR calculation, as follows: "The results are presented as relative risk reduction (RRR) for preterm, term, and all PE, based on a comparison between the modelled PE event rates under antihypertensive therapy and the observed PE event rates" (p9).

Figure 1 (a and b): These Figures are difficult to compare because they use different scales. Visually, they appear to be the same graph. A more helpful approach would be to keep the same scale for both, which would allow a more precise visual comparison of the different risk levels.

Response: Thank you for this feedback. We struggled with this presentation, as one would not be able to see the low-risk data if we used the same scale as the high-risk. We have now combined the figures into one, Figure 1. We are happy to adjust further based on additional feedback.

Minor:

Typo (word missing) in " This means that women with chronic hypertension would be more often if their dBP were >85mmHg"

Response: Thank you for spotting this error. This has been corrected, as follows: "This means that if their dBP were >85mmHg (whether or not on antihypertensive therapy), more women with chronic hypertension would be treated with antihypertensive and those on treatment would receive more medication, as their target BP would be lower (80 or 75mmHg)" (p9).

The Tweetable comment is overstating the findings. This study is not able to say anything directly about the causal association between medication to lower BP and PE.

Response: Thank you for this feedback. We have revised the statement, as follows: "Lower BP is associated with less pre-eclampsia" (p3).

VERSION 2 – REVIEW

**REVIEWER NAME**	Perera, Rafael
**REVIEWER AFFILIATION**	University of Oxford, Primary Care Health Sciences
**REVIEWER CONFLICT OF INTEREST**	None
**AI DISCLOSURE**	I have used grammarly to help with grammar and typos.
**DATE REVIEW RETURNED**	09-Dec-2025

**GENERAL COMMENTS**	bmjmed-2025-001631 Modelling lowering of elevated blood pressure in pregnancy to reduce pre-eclampsia<br><br>Stats Review<br><br>Many thanks to the authors for their responses to my queries. The paper is easier to read and has a clearer message. There are some minor points (including typos and small mistakes) that should be addressed before publication can be recommended.<br><br>Issues:<br><br>The authors point out that for their Table 2 - 'The expected number is calculated separately for each gestational age at birth category of PE, so the total of these categories is not necessarily the same as the 'all PE' expected number of cases.'<br><br>I do not understand why this is the case. Please could you expand/explain? It could be a small example in the Supplementary materials. It is clear, for example, that the number for observed 'all PE's is the same as the sum of the observed Preterm PE <37 weeks + Term PE>=37 weeks. Why is this not the case for the numbers calculated based on the model?<br><br>Two of your RRR in Table 2 (and in the text) are incorrect for 'All PE'. These are for the in pregnancies with dBP >90mmHg and for 'In group with dBP >75mmHG. The numbers presented have been incorrectly copied from cells above. The NNTB are correct. Please correct in the Table and in the rest of the text.<br><br>Please provide a full explanation of what is presented in the Calibration plots. Just expand on what each plotted distribution represents to make it easier for readers to follow.

	Typos:<br>‘Only RAAS [renin-angiotensin-aldosterone system] blockers {ARE} contraindicated for use in pregnancy, based on their fetotoxicity.’<br><br>‘more women with chronic hypertension would be treated with antihypertensive{ES} and those on treatment would receive more medication’

VERSION 2 – AUTHOR RESPONSE

Reviewer: 3

Prof. Rafael Perera, University of Oxford

Comments to the Author

bmjmed-2025-001631 Modelling lowering of elevated blood pressure in pregnancy to reduce pre-eclampsia

Stats Review

Many thanks to the authors for their responses to my queries. The paper is easier to read and has a clearer message. There are some minor points (including typos and small mistakes) that should be addressed before publication can be recommended.

Response: Thank you for your careful review of our work. We believe that we have addressed all issues raised.

Issues:

The authors point out that for their Table 2 - ‘The expected number is calculated separately for each gestational age at birth category of PE, so the total of these categories is not necessarily the same as the ‘all PE’ expected number of cases.’

I do not understand why this is the case. Please could you expand/explain? It could be a small example in the Supplementary materials. It is clear, for example, that the number for observed ‘all PE’s is the same as the sum of the observed Preterm PE <37 weeks + Term PE>=37 weeks. Why is this not the case for the numbers calculated based on the model?

Response: Thank you for this comment and for the opportunity to clarify this point. The FMF competing risks model generates personalised distributions of gestational age at delivery with PE, which allows calculation of the risk of delivery with PE before any given gestational age. When estimating the incidence of each outcome, we calculated outcome-specific risks; for example, the incidence of PE <32 weeks is estimated using the risk of PE <32 weeks. The expected numbers of preterm and term PE do not sum to the total number of PE cases because they are each derived from different risk estimates. Although it would have been possible to estimate the expected number of term PE by subtracting the expected number of preterm PE from the expected number of all PE, we instead used outcome-specific risks for each category. This approach was chosen to optimise risk calibration for each outcome. We have now clarified that we use multiple risks in the methods section, as follows (with new next in blue): “The FMF competing risks model was used to calculate the expected risk of any PE, PE <32 weeks, PE <34 weeks, PE <37 weeks and PE ≥37 weeks, for each pregnancy, and the total expected number of PE cases for the whole population was derived by summing the decimal risks” (p9).

Two of your RRR in Table 2 (and in the text) are incorrect for ‘All PE’. These are for the in pregnancies with dBP >90mmHg and for ‘In group with dBP >75mmHG. The numbers presented have been incorrectly copied from cells above. The NNTB are correct. Please correct in the Table and in the rest of the text.

Response: Thank you for catching these errors, for which we apologise. These have been corrected, in the abstract (p3) and Results text (p11-12) and Table 2.

Please provide a full explanation of what is presented in the Calibration plots. Just expand on what each plotted distribution represents to make it easier for readers to follow.

Response: As suggested, text has been added to the legend of Figures S1 (which applies to Figures S1a-S1e), to provide further clarity, as follows: “Legend: The solid grey line is the line of equality, which represents perfect calibration. The overall incidence is shown by the horizontal interrupted line, and the histograms show the distribution of risks in pregnancies with PE (red) and those without (grey). Calibration intercept and slope are presented alongside AUC”.

Typos:

‘Only RAAS [renin-angiotensin-aldosterone system] blockers {ARE} contraindicated for use in pregnancy, based on their fetotoxicity.’

‘more women with chronic hypertension would be treated with antihypertensive{ES} and those on treatment would receive more medication’

Response: These typos have been corrected, thank you.
